# Pathology and Practice of Medicine

**Published:** 1861-03

**Authors:** Richard J. Dunglison

**Affiliations:** One of the Physicians to the Pennsylvania Institution for the Instruction of the Blind; Honorary Local Secretary to the Sydenham Society of London, etc.


### PATHOLOGY AND PRACTICE OF MEDICINE.

BY RICHARD J. DUNGLISON, M.D.,

ONE OF THE PHYSICIANS TO THE PENNSYLVANIA INSTITUTION FOR THE INSTRUCTION OF THE BLIND; HONORARY LOCAL SECRETARY TO THE SYDENHAM SOCIETY OF LONDON, ETC.

I.—*Clinical Report on Pneumonia, based on an Analysis of* 133 *Cases.* By AUSTIN FLINT, M.D., Professor of Clinical Medicine and Pathology in the New Orleans School of Medicine, etc. (American Journal of the Medical Sciences, January, 1861, pp. 16–55.)

Professor Flint's clinical records, for the past twelve years, contain 133 cases of pneumonia, (exclusive of cases in which the patients were under five years of age,) 49 of which were observed in the Buffalo Hospital of the Sisters of Charity, (1848–1859;) 53 in the New Orleans Charity Hospital, (1858–9 and 1859–60;) 11 in the Louisville Marine Hospital, (1853–4 and 1855–6;) and 20 in private practice, (1851–1860,) mainly at Buffalo. The plan of analysis has reference to the ages, occupations, and habits of the patients, the circumstances connected with the causation of the disease, etc. Upon each of these points the statistics recorded by so reliable an authority on the subject of thoracic affections, are so well worthy of permanent record, that we here make an abstract of the valuable information which the paper contains.

I. *Age.*—Pneumonia has no predilections for any particular periods of life. The number of cases occurring at different ages is sufficient to show that no period of life is exempt from it.

II. *Sex.*—The male sex is much oftener affected than the female—of eighty cases in hospitals and private practice, but ten being females.

III. *Occupation.*—It is not produced directly by any particular occupation; occurs chiefly among persons whose callings involve muscular exertion and exposure to the weather. Occupation noted in eighty-seven cases, exclusive of female patients—fifty-two of these were laborers. Occupations of the remainder of a kind to involve, more or less, exposure to the weather.

IV. *Causation.*—It has no pathological connection with periodical fever. Other pulmonary affections do not predispose to it; some, as asthma, emphysema, and chronic pleurisy, appear to afford a protection against it; occurs, but not frequently, in those affected with organic disease of the heart; may be produced traumatically, by external injury, or the passage of a pistol-ball through the chest; not one of the secondary affections referable to degenerative disease of the kidney. May not infrequently be traced to some exciting cause, as unusual exposure to cold and wet, but generally occurs when no exciting cause is apparent; is oftener developed abruptly than in a gradual manner.

V. *Comparative frequency of cases of the disease at New Orleans, Louisville, and Buffalo.*—Cases not as numerous at Buffalo as at Louisville, and by far more frequent at New Orleans than in the other two places. At Buffalo cases occur in January, March, and April, much more frequently than at other parts of the year; at New Orleans, more numerous in November and December than in any other of the winter months; and of the comparatively few cases observed at Louisville, three-fourths occurred in January.

It would be interesting to see if these irruptions of pneumonia are connected with any uniform meteorological changes, but the data for such a comparison are not at present available. Still another fact leading to the conclusion that pneumonia is determined by external causes, is its occasional prevalence as an epidemic in southern and southwestern portions of our country, especially among the negro population.

VI. *Seat of the disease and extent of lung affected.*—Results in 121 cases show the almost invariable extension of the inflammation over at least a lobe of the lung; that the most frequent variety of lobar pneumonia is that in which the inflammation extends over the whole of the right lung, (37 cases.) This would not have been expected, and would not be the fact were it not for the tendency of the disease to extend over the whole of the right lung being greater in New Orleans and Louisville than in Buffalo. Next in order of frequency is that variety in which the lower lobe of the right lung is alone affected, (29 cases.) And at Buffalo the latter is the most frequent variety.

These results, however, show that the cases in which the disease is limited to the lower lobe of the left lung, (25 cases,) are nearly as numerous as those in which the lower lobe of the right lung is alone affected. This would not have been expected, the common belief being that the lower lobe of the right lung is much oftener the seat of pneumonia. Inflammation seated primarily in the lower lobe of the left lung rarely extends over the whole of that lung, (9 cases.) The lower lobes of both lungs (double pneumonia) are rarely affected, (7 cases.)

Pneumonia very rarely attacks primarily the upper lobes, and oftener the upper lobe of the right (8 cases) than the upper lobe of the left lung, (3 cases.) When the whole of either lung is affected, the liability of extension of the disease to the opposite lung is excessively small.

VII. *Complications of Pneumonia, and the Occurrence of Gangrene and Abscess.*—1. *Pericarditis and Delirium Tremens* were complications oftenest observed, each occurring in 8 out of 133 cases. The latter is simply a superadded affection, generally attributable in part, or entirely, to discontinuance or diminished use of alcohol after the attack of pneumonia. Six of the eight cases of pericarditis and three of the eight cases of delirium tremens were fatal. These results show the gravity of pneumonia complicated with pericarditis, but recovery may take place even when, in addition, the inflammation of the lung eventuates in abscess.

Exclusive of pericarditis and delirium tremens, complications of pneumonia are by no means common—a remark applicable to New Orleans, Louisville, and

Buffalo; but complications exist more often at New Orleans and Louisville than at Buffalo, and consequently greater gravity of the disease.

2. *Gangrene and Abscess.*—In four cases existence of abscess was ascertained ; may have existed in some fatal cases in which it was undetermined, the bodies not being examined after death. The pneumonia eventuated in gangrene of the lungs in one case only.

VIII. *Fatality and Duration.* 1. *Fatality.*—In 133 cases there were 35 deaths, a fatality of a fraction over 26 per cent. Proportion of fatal cases considerably larger at New Orleans and Louisville than at Buffalo, the ratio being, at the latter place, a fraction over 17 per cent.; at New Orleans, a fraction over 32 per cent.; and at Louisville, a fraction over 63 per cent. Of the 35 fatal cases 16 were either complicated or associated with other important affections.

The proportionate fatality in uncomplicated cases was 19 in 112, or a fraction under 17 per cent. Pneumonia uncomplicated and not associated with any other important affection, if limited to one lobe, is not a disease dangerous to life, since there were but two examples among the 133 cases analyzed.

Under this head arise four questions :—

*a. Of complicated cases what proportion were fatal?*—Of the whole number in which other affections became developed in the course of the pneumonia, (21,) 11 were fatal. Of 7 cases in which pulmonary tubercles coexisted, recovery took place in all save one.

*b. In how large a proportion of cases in which inflammation extended over more than one lobe did death take place?*—Of 11 cases of double pneumonia, the number of deaths was 8 ; of 57 in which it extended over two or more lobes, 19 died, the proportion being exactly one-third. The fatality is thus seen to be greatest in double pneumonia. Next in order are cases in which the whole of the right lung is affected. The fatality is strikingly less when the whole of the left lung is affected, being only as one to nine.

*c. Does age exert any influence on the fatality of this disease?*—In the largest number of cases in the whole collection the ages were between 20 and 30, while in the largest number of fatal cases they were between 30 and 40. Age does exert an influence on the fatality, the liability to a fatal result being greater in proportion as patients approach the age of sixty—this collection containing no examples of a greater age than sixty years.

*d. Does intemperance exert an influence on the fatality?*—It is probably true that habits of drinking were noticed in a larger proportion of the cases in which they existed, than the absence of these habits in the cases in which the patients were temperate. Hence the results do not fairly exhibit the influence of intemperance.

2. *Duration.*—In 30 cases the shortest duration from the date of the attack to the time when the patient could be pronounced convalescent was 5 days, and the longest 23 days. The mean duration was a fraction over 12 days.

In 14 of the fatal cases the shortest duration from the date of the attack to the time of death was 3 days, and the longest 20 days. The mean duration was a fraction over 10 days.

The duration in hospital cases, from the date of admission to the time of discharge, varied from 4 to 70 days. The length of stay in hospital, however, in a series of cases, does not show the length of time that the patient was necessarily detained by the disease.

IX. *Treatment.*—The objects of treatment have been the palliation of symptoms, placing the system in a condition of tolerance as regards the disturbance caused by the local affection, and sustaining the vital powers so as to obviate a tendency to death by asthenia or exhaustion, and to promote a speedy and complete recovery.

1. *Points of inquiry in regard to treatment.*—The proportionate fatality in cases in which different therapeutical measures were employed, the duration of the disease in cases differently treated, the immediate apparent effects of remedies, the almost impracticability of arresting pneumonia, and its intrinsic tendency to death or recovery, when left to itself—*i.e.* without treatment.

2. *Progress without treatment.*—A few cases in this collection illustrate the favorable progress of the disease and recovery without medical treatment, and even under most unfavorable hygienic circumstances. Recovery, favorable progress of the disease, and a brief duration, even if an entire lung be affected, do not necessarily constitute evidence of the curative efficacy of any plan of treatment which may have been pursued.

But, on the other hand, taking into view the fact that in only two cases in which the disease was uncomplicated and limited to a single lobe, did it prove fatal, a considerable number of fatal cases, when no important complication existed, and the disease did not extend to two or more lobes, should be considered as furnishing good grounds to suspect that the treatment pursued contributed to the fatality.

3. *Remedial measures. a. Blood-letting.*—So far as any conclusions are admissible from the facts, they show that bleeding did not prevent extension of the disease to the entire lung, which occurred in 5 of the 12 cases; nor the affection of the lower lobes of both lungs, which occurred in 1 case; nor the development of delirium tremens, which occurred in 1 case; nor pericarditis in 1 case.

It did not diminish the fatality, which was at the rate of 36 per cent., being considerably larger than the rate in all the cases, (26 per cent.;) much larger than that at Buffalo, (17 per cent.;) somewhat larger than at New Orleans, (32 per cent.;) but falling below the rate at Louisville, (63 per cent.)

The analysis of cases in which blood-letting was employed furnishes no evidence that this measure affected, either favorably or unfavorably, the progress of the disease. In some of the cases it appeared to afford relief.

The longest duration of these cases, from the date of the attack to convalescence, was 30 days; and the next longest 15 days. The shortest duration was 9 days, and the next shortest 11 days. The mean duration was a fraction under 15 days. The mean duration of stay in hospital was a fraction under 25 days. The average duration from the attack to convalescence is exactly the same as in the aggregate of the other cases mentioned here, viz., a fraction over 12 days.

*b. Tartar emetic* was prescribed in 12 cases, in small doses, (gr. $\frac{1}{16}$ or $\frac{1}{8}$,) and nearly always given in conjunction with opium or the sulphate of morphia in small doses. Palliation of certain symptoms was alone looked for through its sedative influence on the circulation and nervous system. It is fair to presume that the antimony exerted in several cases a certain amount of palliative influence. It was not given under circumstances likely to render a depressing remedy injurious, either directly or indirectly, by conflicting with supporting measures.

*c. Sulphate of quinia* entered more or less into the treatment in 32 cases; in 9 in doses of gr. ij, three times daily, generally in conjunction with opium in small doses, brandy, and sometimes carbonate of ammonia; in nearly all of the remaining 23 cases, in doses of gr. v, three times daily, continued for a variable period. Of the 23 cases, 3 only were fatal; all those which recovered, save 3, were uncomplicated, and the inflammation was limited to a single lobe in all the other cases save one.

It is doubtful whether any inference is to be drawn from the fatality, except that the sulphate of quinia did not exert an unfavorable influence on the termination of the disease. The immediate apparent effects were, in several instances, favorable in a marked degree; the pulse, in several cases, diminished in frequency notably under its use, a corresponding reduction in the frequency of the inspirations sometimes taking place and sometimes not.

*d. Opium.*—Opiates entered more or less into the treatment in 100 cases, and were either the only, or the most important, remedy in 49. In 46 of these 49 cases, the sulphate of morphia was given in doses of gr. $\frac{1}{4}$ or upwards, or opium in doses of gr. ij or upwards, every 4 or 6 hours. Of the 46 cases treated with full doses of opium, 11 were fatal, a fraction over 23 per cent. This is somewhat under the fatality, in the whole number of cases analyzed, which was a fraction over 26 per cent.

So far as any conclusion from the fatality is admissible, it is certainly favorable to the treatment of the disease with full doses of opium, as compared with other methods of treatment pursued in the cases collected by Dr. Flint. Among the cases treated with full doses of opium, ending in recovery, in 12 the inflammation extended over a whole lung, or 30 per cent.; while the proportion in 110 cases treated by different methods, and including fatal cases as well as those which recovered, was only a fraction over 31 per cent.

Full doses of opium, however, do not afford a protection against the extension of the disease beyond a single lobe. In 22 of the 35 cases of recovery, the mean duration was a fraction over 11 days.

In the cases in which other remedies were added, (excepting alcoholic stimulants,) they were subordinate to the opium treatment. Dr. Flint has never observed more than slight narcotism produced by large quantities of the remedy, when judiciously given, a fact which shows that pneumonia involves tolerance of full doses of opium.

*e. Alcoholic stimulants,* given more or less freely, and continued for a greater or less period during the progress of pneumonia, produced no apparent unfavorable effects. The facts show that, given in conjunction with opium and nutritious diet, they affect favorably the progress of the disease and diminish its fatality, and are not contraindicated by the coexistence of pericarditis or organic disease of the heart.

In conclusion, it has been recently stated that the chlorides disappear from the urine during the progress of the exudation in pneumonia, and reappear so soon as the exudation ceases. Hence, it is considered by some that the absence of chlorides is evidence that the inflammation is extending; while, on the other hand, their reappearance is a test that the inflammation has reached the limit of its extension. The conclusions arrived at by Dr. Flint, from the examination of eleven cases, are, that the absence of the chlorides may be considered as evidence that the exudation is going on, but the presence of the chlorides does not constitute conclusive evidence that exudation is not going on.

II.—*The Last Sickness of General Washington, and its Treatment by the Attendant Physicians.* By JAMES JACKSON, M.D.

Attached to the Life of Washington, by Mr. Everett, is a memoir, drawn up by Dr. James Jackson, of Boston, upon the last illness of that distinguished man. In the language of the writer, "the circumstances of his short disease, its character viewed scientifically, and the treatment adopted by his physicians, have all been ascertained and discussed; and the remedies employed have been spoken of by some persons in terms of strong reprobation."

Though complaining of sore-throat and hoarseness on the 12th of December, 1799, it was not until the following night that the symptoms of Washington's illness became severe. He then had a chill, followed by great difficulty in breathing, speaking, and swallowing, symptoms which continued until the following night, that of his death. The breathing caused him most distress; the more because his strongest efforts were insufficient to supply his lungs with as much air as his system had need of. He was in fact strangulated by the closure of the windpipe, as much as if a tight cord had been twisted around his neck.

*Acute laryngitis* is rare; it had not, at the time of Washington's death, been clearly described, so as to be distinguished from other diseases about the throat. About 1810 it was first brought into notice, and distinctly described by Dr. Matthew Baillie, of London. After detailing for the popular understanding pathological changes occurring during an attack of acute laryngitis, the causes of obstruction of the upper parts of the respiratory and digestive apparatus, and the mode of death, Dr. Jackson proceeds to consider the treatment of this alarming affection.

It has been thought by many persons, medical and non-medical, that General Washington was not treated judiciously; and some, perhaps, believe that, by a different treatment, his life might have been preserved. He was bled, he was blistered, and calomel and antimony were administered internally. The best teachers at the present day recommend bleeding and blistering. In addition, the English teachers advise the use of mercurials carried to the point of salivation, and our own did the same until very lately. Some of them, perhaps, do it now. Some, if not many, would add antimony and opium to the calomel, or other preparations of mercury.

The chance of success of blood-letting in acute laryngitis is great in the very beginning of the inflammatory process; but it is less the later the period at which the remedy is employed. There is not, however, any other measure by which effectual relief is so likely to be produced. But who would advise the active treatment requisite for this purpose in every case of a *hoarse cold*, which is the first stage? There is no doubt that every discreet man would choose to incur the slight hazard of the severe disease, rather than resort to a copious bleeding every time he had a hoarse sore-throat.

In Washington's case, the first stage was of short duration. Bleeding was resorted to early, by his own direction. But that bleeding was nominal. His wife objected to it, because the patient was old, and it had not been prescribed by a physician. When Dr. Craik reached him, some hours afterward, he prescribed a new venesection. Calomel and antimony were given him, but there is not any reason to believe that they were given in large doses; though Dr. Craik and his coadjutors have been reproached on this score. In 1799 the use of mercurials in inflammatory diseases was very rare in Great Britain, though it was very common in this country. At the present day, the reverse is true.

We fully concur with Dr. Jackson in his defense of the reputation of Dr. Craik and his medical friends, that they did as well, at least, as any of their critics would have done in the like case.

III.—1. *Observations on Favus.*—By WILLIAM PIRRIE, JR., M.D., late Assistant Surgeon H. M.'s 71st Highland Light Infantry. (London Lancet, Dec. 15, 1860.)

2. *A Clinical Report on True Ring-worm, (Tinea Tonsurans.)* By JONATHAN HUTCHINSON, Surgeon to the Metropolitan Free Hospital, etc. (Medical Times and Gazette, Jan. 12, 1861.)

By an opportune coincidence, two of the English journals contain investigations into the nature and history of two forms of cutaneous disease, from which we abstract the following conclusions:—

| FAVUS. | TINEA TONSURANS. |
| --- | --- |
| 1. Favus is essentially characterized by the presence of a fungus, which is easily discovered by the microscope. | 1. True ring-worm, or tinea tonsurans, may be defined as a disease affecting either the scalp or the general surface, in which circular patches are formed, on which the hairs break off short, and a slight branny desquamation is seen, both hairs and epidermic scales exhibiting under the microscope the sporules and thalli of a fungus. |
| 2. It is peculiar to the young, and confined to the poor and destitute. | 2. Ring-worm on the scalp is rarely seen, excepting in children; but on the general surface is not very unfrequent in young adults. |
| 3. It is most commonly met with on the scalp, but occasionally on other parts of the body. | 3. It is contagious, and spreads by contagion only. |
| 4. The hair follicles are only secondarily affected. | 4. It is not attended by any particular form of dyscrasia, but, on the con- |
| 5. It is by no means a rare disease in Scotland, being exceedingly common in Edinburgh, and having been more so for several years past in Aberdeen than in Glasgow. | |
| 6. It is generally considered more common in Ireland than in England. | |

7. It is a blood disorder, and the fungus is not the sole nor the original cause of the eruption.

8. Many are unsusceptible to it; it is feebly contagious, and very often arises independently of contagion.

9. The previous state of health has an important bearing on its outbreak.

10. It is intimately connected with the strumous diathesis.

11. Want of cleanliness strongly predisposes to it.

12. For its removal, general as well as local treatment is necessary.

trary, often attacks children in perfect health.

5. It is much more easily curable on the general surface than on the scalp, owing to the circumstance, that in the latter situation the fungus has obtained access to the follicles of the hairs.

6. Being a purely local disease, ring-worm does not require, *per se*, any constitutional treatment.

7. A purely local treatment, if efficiently pursued, is always and rapidly successful.

8. Epilation, and the use of one or other of the known parasiticides, are the measures of treatment required.

9. There is no real difference between ring-worm on the scalp and ring-worm on the general surface.

10. Ring-worm, although not unfrequently causing minute vesicles, has no true analogy with herpes.

IV.—*Patency of the Foramen Ovale of the Heart Producing Two Opposite Classes of Symptoms.* A Lecture delivered at the Grosvenor Place School of Medicine. By DR. RICHARDSON. (Medical Times and Gazette, December 22, 1860, p. 605.)

The history of a case of cyanosis, complicated with tubercular disease of the lungs, in a child five years of age, afforded Dr. Richardson an opportunity of discussing the pathology of the affection. After death consequent on symptoms of asphyxia, terribly labored breathing, and marble coldness of surface, he detected three abnormal conditions, viz.: 1. Each lung was literally charged with tubercles of the size of millet-seeds, which did not involve any neighboring structures in purulent degeneration. 2. The blood was fluid, but of a dark, venous color, both in the venous and arterial side of the circulation. 3. The heart was enlarged on the left side, the right auricle distended, the right ventricle small, the left auricle a little smaller than natural, the left ventricle large; the original foramen ovale in the septum auriculorum was split into three openings.

The foramen ovale is thus left open, on account of the deficient formation of the membrane, which, after birth, ascends from the lower margin of the foramen and closes up, in the majority of cases, the foramen altogether; and for the first few days of life the circulation is carried on normally with a distinct opening between the auricles. The continuance of the patency is owing, in cyanosis, to the persistence of the current of blood from the right to the left auricle, due to a constant obstruction in the lungs. Death, in cyanosis, ordinarily occurs from pulmonary congestion, cyanotic patients being almost exempt from inflammatory disorders, though suffering readily from every depressing influence. In the present case the tubercular deposit can scarcely be attributed directly to the cyanosis; yet the activity of the phthisical symptoms was modified by the cyanotic state, the hectic suppressed, and the local development of inflammatory products around the tubercular masses prevented. The heart sounds, as usual, were normal.

Arterial blood may pass from the left auricle into the right, as is described in several cases given by Dr. Richardson. In one, described by Dr. Mayne in the Dublin Journal for 1848, the skin was pale, not blue, moist constantly, not dry and cold. Exercise produced embarrassment and even death; and the mental faculties were reduced to the lowest possible manifestation. In Dr. Richardson's case, the brain, fed always with blood largely impure, was active; in Dr. Mayne's case, the brain, fed always with arterial blood, was inert. No venous blood found its way into the arterial capillaries; hence cyanosis was absent.

The left ventricle being small and the aorta contracted, and the right ventricle large and the pulmonary arteries and veins dilated, there was always a small systemic with a great pulmonic current. While the patient received no excess of oxygen, the blood in the lungs only being capable of taking up a certain amount, she must have exhaled at all times a minimum quantity of carbonic acid; and hence those systemic changes leading to the formation of carbonic acid, and so essential to healthy nutrition, being suppressed, languor and depression were always present. Embarrassed breathing, sometimes occurring, would be referable to the temporary congestion of the lungs, which ensued whenever the right side of the heart was stimulated to undue activity.

Two opposite classes of symptoms are thus seen to arise from the same structural deficiency.

V.—*Diphtheria; its Nature and Treatment, with an Account of the History of its Prevalence in various Countries.* By DANIEL DENISON SLADE, M.D., of Boston, Massachusetts. The Dissertation to which the Fiske Fund Prize was awarded, July 11, 1860. (American Journal of the Medical Sciences, January, 1861, pp. 145–174, and 301–326.)

We have already furnished an abstract of one important paper from the pages of the American Journal, and we now make an analysis of another, which is a carefully prepared digest of the principal points in relation to diphtheria. After giving Bretonneau's description of the disease, that "nothing is diphtheria that is not a pellicular exudation, no such exudation is diphtherical which is not capable of acting as a virus or contagion," Dr. Slade remarks that it is only by a comparison of the various epidemics of "sore-throat" which have prevailed at intervals in various parts of the world, that we can ascertain how far Bretonneau's description is to be taken as a model of the disease. He therefore gives an account of those which had prevailed in various parts of Europe, and compares the descriptions of different writers upon these epidemics with that of Bretonneau, and then shows that the latter was incorrect in denying the presence of all constitutional disturbance, as also in insisting upon the absence of all relation between diphtheria and gangrene of the fauces—both of these conditions having been frequently observed, particularly during the epidemics of late years.

Bretonneau's idea of croup, which he associates with diphtheria, does not conform to the present views of that disease, founded as they are upon the description given by Dr. Home.* The distinctions between diphtheria and croup are dwelt upon, as also the non-identity of diphtheria and scarlatina.

A comparison of the descriptions of the disease by various writers, as it appeared in the several counties of England, gives no marked uniformity, and but little correspondence with Bretonneau's model. Dr. Slade then considers diphtheria in America, and gives at some length a description of an epidemic of "sore-throat," by Dr. Bard, also an account of the epidemics in California and other parts of the Union. All these epidemics of "sore-throat" are connected by a bond of union to be found in the pathological anatomy of the disease, which consists in the peculiar exudation.

Although Bretonneau fully recognized this fact, his description was deficient; therefore those of MM. Barthez and Rilliet are subjoined as being more comprehensive. The disease is also considered as existing under two forms, the mild and severe.

The nature of the disease is then taken up; first, the characteristics of the false membrane, its physical appearance, its seat, the experiments of Bretonneau in order to ascertain the specific nature of the diphtheritic membrane, its microscopic appearances, and its dependence upon certain parasites.

Is diphtheria infectious? Having given the arguments of various authors, Dr. Slade replies, that although we are ignorant of the exact laws which governed these epidemics, we *could* find certain hygienic or individual circumstances which undoubtedly had their effect, either as direct or as predisposing causes. He thinks further observation necessary before ascribing any settled prognostic value to the presence of albumen in the urine. He then refers to after-effects of the disease, especially upon the nervous system.

It is only within the last few years that anything like unanimity in the treatment of diphtheria has prevailed. It is now universally considered as an asthenic disease, and consequently will bear no depletory measures, but, on the contrary, requires tonics, stimulants, and a nourishing diet, even in the early stages. Blisters, leeches, and local bleedings of any sort should be prohibited. The tonics best suited are enumerated. The auxiliary measures first spoken of are the local applications to the fauces; their utility and propriety, and the various agents which have been employed, and the mode of using them are referred to. The ablation of the tonsils recommended by Bouchut is inadmissible, excepting under rare circumstances.

Tracheotomy is discussed at considerable length. Remarking that the two diseases, inflammatory croup and diphtheria, were on an equal footing as regards the applicability of the operation; the various objections brought against it, as the small amount of success, the difficulties of performing it, the tendency to the production of bronchitis, etc., are all considered and answered. The proper time for performing the operation is decided to be an intermediate period. Some

---

* Inquiry into the Nature, Cause, and Cure of the Croup. Edinburgh, 1765.

necessary rules are given as to the size of the canula, the state of the surrounding atmosphere, the importance of having some competent person at hand in case of emergency, the propriety of keeping up the medical treatment, and the time for removing the canula.

Having given a summary of the treatment recommended by some of the leading men in Europe, the article concludes by a brief consideration of the operation for "tubing the larynx."

VI.—*Acute Pericarditis with Effusion; Recovery with Adhesion of the two Surfaces of the Pericardium; Absence of any Rheumatic Affection.* A Case in King's College Hospital, under the care of Dr. Beale. (Archives of Medicine, No. 6, 1860.)

It is very rare to meet with cases of acute pericarditis with effusion occurring independently of rheumatic fever. In a case in the medical ward of King's College Hospital the attack appears to have originated in a cold from exposure; but throughout the whole course of the malady there was no indication of rheumatic affection. All the symptoms from which he suffered appear referable to the heart affection. The amount of the effusion in the pericardium was very great. The œdema of the legs depended probably upon an impediment to the return of blood to the heart, created by the pressure exerted by the distended pericardium.

The treatment resorted to consisted chiefly of counter-irritation by turpentine stupes, linseed-meal poultices over the heart, ammonia and chloric ether, with a moderate quantity of brandy, (two to four ounces in twenty-four hours.) Had the patient been very restless, opium would have been given, and mercurial ointment would have been rubbed in over the heart; but as he was carefully watched from day to day, it was evident that he was progressing as satisfactorily as could be expected, and it was therefore thought better to adhere to the treatment above described, believing that the fluid would be gradually absorbed, if the patient's strength and nutrition were properly kept up. This man was under treatment less than four weeks, and the whole of the fluid effused into the pericardium, which at one time must have been very considerable, was probably reabsorbed.

The most favorable result which could be hoped for in such a case, the adhesion of the two surfaces of the pericardium, certainly occurred. The man left the hospital; but about three weeks afterward, in consequence probably of want of care on the patient's part, the effusion reappeared and the pericardium was again distended to its former size. The fluid was again absorbed under the influence of mercurial inunction, and the patient left the hospital a second time. He still suffers from great difficulty of breathing, his pulse is weak, very quick and fluttering, and he is quite unable to work.

